# *Sommera cusucoana*, a new species of Rubiaceae from Honduras

**DOI:** 10.3897/phytokeys.57.5339

**Published:** 2015-10-13

**Authors:** David H. Lorence, Anke C. Dietzsch, Daniel L. Kelly

**Affiliations:** 1 National Tropical Botanical Garden, 3530 Papalina Road, Kalaheo, HI 96741 USA; 2 Department of Botany, School of Natural Sciences, Trinity College, the University of Dublin, Dublin 2, Ireland

**Keywords:** Rubiaceae, *Sommera*, Honduras, Cusuco, conservation

## Abstract

*Sommera cusucoana* Lorence, D. Kelly & A. Dietzsch, **sp. nov.**, (Rubiaceae), a new species from Honduras, differs from the other Mesoamerican *Sommera* species by the combination of large, obovate leaves with long red petioles, glabrous or glabrate intervenal areas, red stipules, lax, sparsely pubescent inflorescences with red axes, flowers with red hypanthium and calyx, long fruiting pedicels, and dark red mature fruits. It is known only from the type locality in Cusuco National Park.

## Abstract (es)

Se describe e ilustra *Sommera cusucoana* Lorence, D. Kelly & A. Dietzsch, **sp. nov.** (Rubiaceae), especie nueva de Honduras. Se trata de un árbol que se difiere de las otras especies Mesoamericanas de *Sommera* por sus hojas grandes obovadas con peciolos largos rojos, áreas intervenales glabras o glabradas, estípulas rojas, inflorescencias laxas, escasamente puberulas, flores con hipanto y cáliz rojo, pedicelos fructíferos largos, y frutos maduros rojos. Se conoce sólamente de la localidad tipo en el Parque Nacional Cusuco.

## Introduction

*Sommera* Schltdl. is a genus of Rubiaceae ranging from southwestern Mexico through Central America to South America, usually below 2000 m elevation in evergreen wet forests and riparian forests or less often in drier pine-oak forests (one species). The genus comprises about 10 species of shrubs or small trees characterized by relatively large and soon deciduous paired intrapetiolar stipules; relatively large leaves often strigose-pubescent on the veins beneath, with conspicuously parallel, lineolate minor venation; relatively few-flowered axillary or subaxillary cymes; small externally pubescent flowers; and fleshy 2-locular berries with numerous small, angulate seeds. Although traditionally placed in subfamily Cinchonoideae tribe Mussaendeae (e.g. Borhidi 2006, Dwyer 1980), recent molecular studies indicate *Sommera* belongs in subfamily Ixoroideae, tribe Condamineae (Bremer 2009).

L. O. Williams (1973) reviewed the Central American and Mexican *Sommera* species but failed to provide a diagnostic key or illustrate his four new species. Lorence (1993) described and illustrated *Sommera parva* Lorence, a diminutive new species from Chiapas. In his treatment of the genus for *Flora Mesoamericana* Lorence (2012) recognized six species from the Mesoamerican region (Chiapas and the Yucatán Peninsula to the Panamá/Colombia border). One additional species (*Sommera grandis* (Bartl. ex DC.) Standl.) occurs in southwestern to western Mexico outside the Mesoamerican region, and two more have been described from South America (in Colombia, Peru, and Brazil).

**Figure 1.**
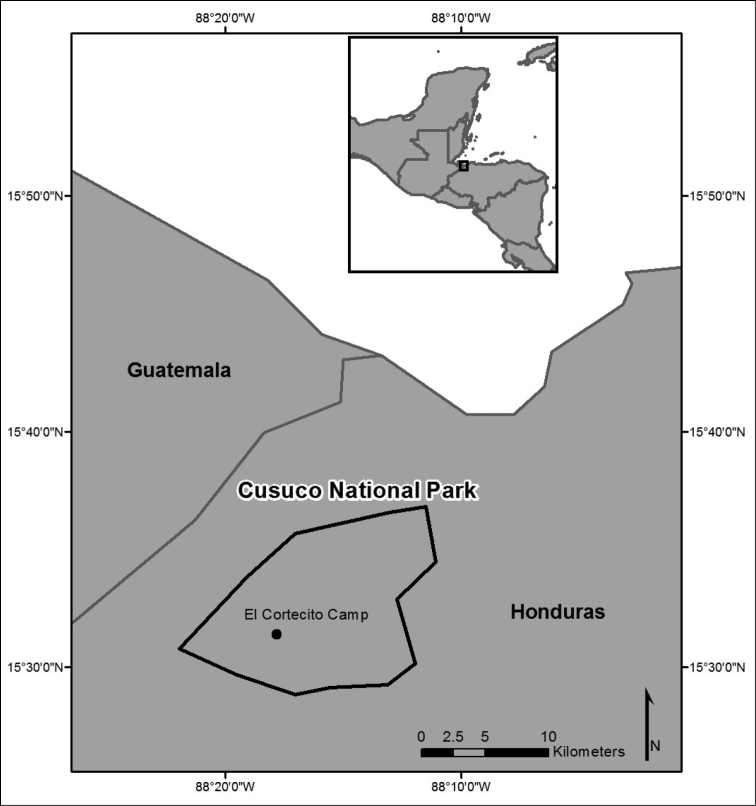
Map showing location of Cusuco National Park. Inset map showing position of Honduras.

During the course of an ecological survey of montane rain forest vegetation in Cusuco National Park in Honduras (Figure 1), a striking new species of *Sommera* was discovered. Although only a single individual was collected, this new species is immediately distinguished from the other Mexican and Mesoamerican *Sommera* species by its red petioles, stipules, inflorescences, hypanthia, fruits and infructescences, and long flowering and fruiting pedicels.

## Results

### Sommera cusucoana

Taxon classificationPlantaeGentianalesRubiaceae

Lorence, D. Kelly & A. Dietzsch sp. nov.

urn:lsid:ipni.org:names:77150371-1

Figures 2 , 3 , 4

#### Type.

Honduras. Prov. Cortes, W of San Pedro Sula. El Cortecito campsite (near left bank of river), Parque Nacional El Cusuco, Sierra de Merendón, UTM 361834 1716534 + 11 m, 1333 m alt., 5 July 2013, D. L. Kelly, A. C. Dietzsch & W. Lopez 15079 (Holotype TEFH!, Isotypes MO and TCD).

#### Diagnosis.

Differs from its congeners by the combination of large, obovate leaves with long red petioles, glabrous or glabrate intervenal areas, red stipules, inflorescences 2–4-flowered, sparsely pubescent, with red axes, flowers with red hypanthium and calyx, and mature fruits dark red with long pedicels.

#### Description.

Tree 10 m high, branchlets 5–6 mm in diam., glabrous, finely ribbed when dry, with sparse, pale +/- ellipsoidal lenticels. **Leaves** opposite, blades 21.5–30 × 10.2–15 cm, obovate, acuminate, finely pointed, cuneate and +/- asymmetrical at base, drying membranaceous, glabrous above, strigillose beneath on costa and 2°–4° veins, intervenal areas glabrous, 2° veins 13–14 pairs, eucamptodromous, venation prominulous; petioles 4.5–9 cm long, sparsely strigillose, red when fresh; **stipules narrowly** deltate to lanceolate, 3–3.8 cm long, when fresh red with thin white margins, glabrous, caducous. **Inflorescences** 5.5–6.5 × 3–7 cm, dichasial, 2–3–4-flowered, axes sparsely strigillose, red when fresh; peduncle 2.5–3.8 cm long, bracts oblong-elliptic, c. 1 mm long, caducous; pedicels 1–2.8 cm long, with bract scars medially. **Flowers** with hypanthium 5 × 3–4 mm, turbinate to obovoid, glabrous; calyx limb red, cupuliform, 2–3 mm long with the tube 0.6–1 mm long, externally glabrous, lobes 5, 1–1.2 × 2 mm, broadly triangular, obtuse, equal, margins densely ciliolate; corolla cylindrical-funnelform, yellowish-cream, fleshy, tube 10–11 × 2.8–3 mm, externally densely hirtellous-tomentose, lobes 5, 1.5 × 3 mm, triangular-ovate, densely papillose-puberulent internally; anthers, style and stigmas not seen. **Fruits** 12–15 × 9–13 mm, subglobose to broadly ellipsoid, dark red at maturitiy, glabrous. **Seeds** numerous, 1–1.8 mm long, irregularly polygonal-angulate, testa dark brown, reticulate. (Figures 3, 4).

**Figure 2.**
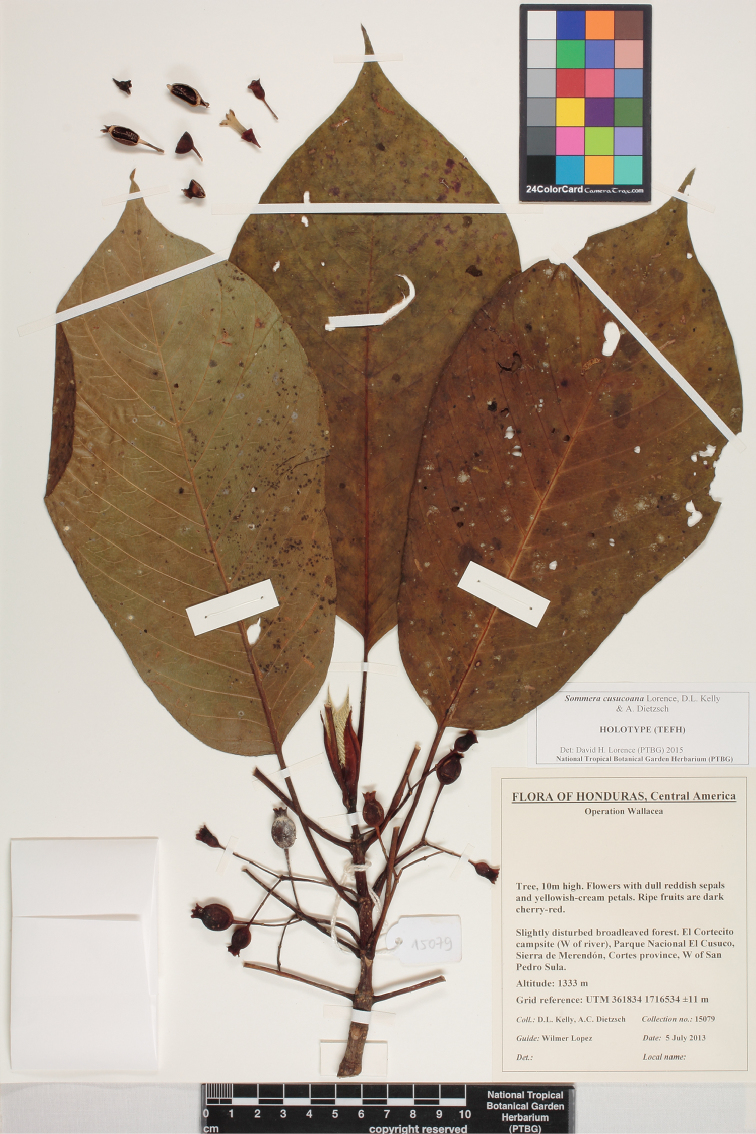
Scan of Holotype specimen of *Sommera cusucoana* (to be deposited at TEFH).

**Figure 3.**
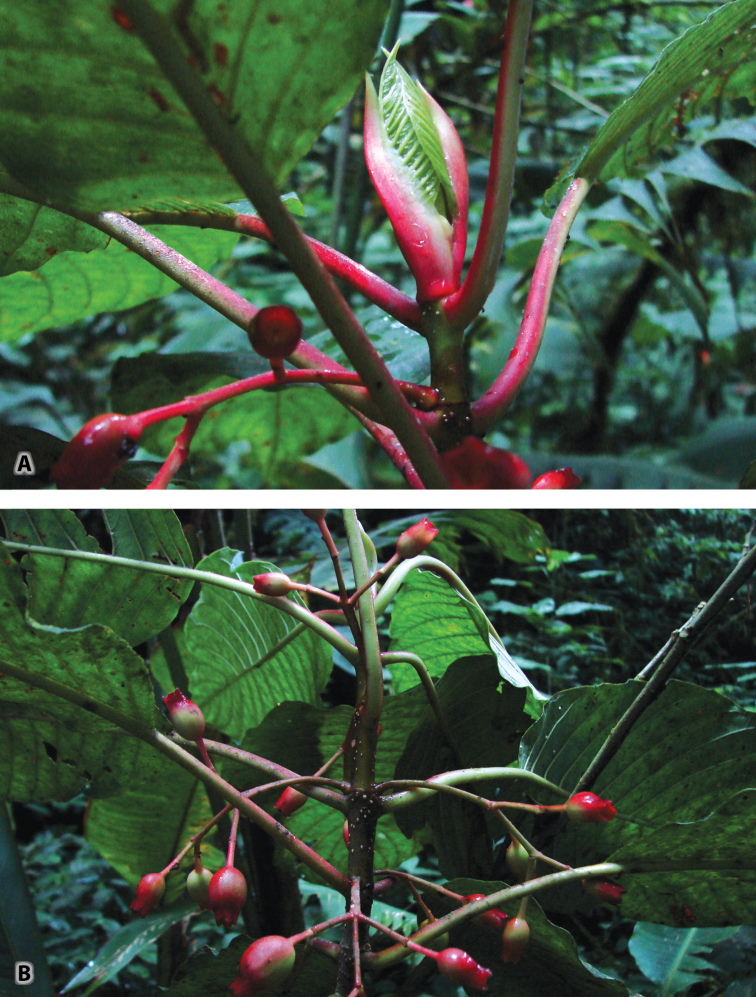
*Sommera cusucoana*. **A** Tip of shoot with infructescence and leaf pair emerging between pair of stipules. Note red color of stipules, petioles, and infructescences **B** Freshly cut branch with inflorescence and infructescences. Photos by A.C. Dietzsch, 5 July 2013.

**Figure 4.**
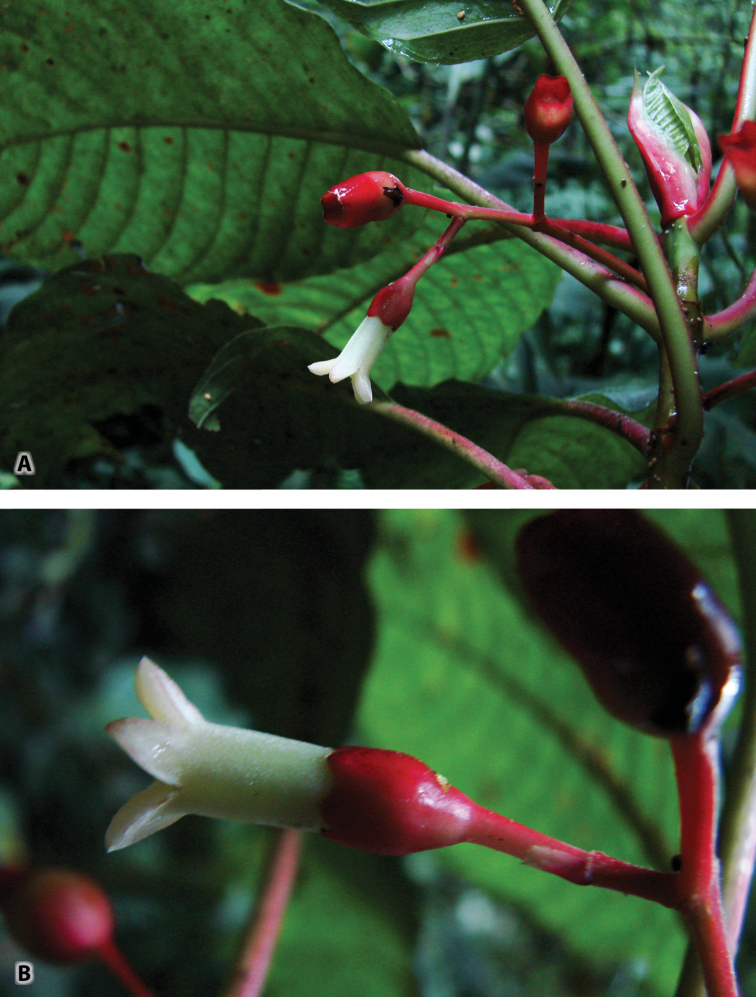
*Sommera cusucoana*. **A** Tip of shoot with flower, developing fruits, and leaf pair emerging between pair of stipules **B** Flower at anthesis and developing fruit (appearing dark because in shadow). Photos by A.C. Dietzsch, 5 July 2013.

#### Habitat and ecology.

Only two individual trees were located, about the same size and within a few meters of each other. The site is within Cusuco National Park, in the upper slopes of the Sierra del Merendón. These upper slopes (highest point 2242 m) are largely covered by montane rain forest vegetation. The bedrock is composed of a mixture of gneiss and schist (The Nature Conservancy et al. 1994); slopes are steep and soils are strongly acidic (D.L. Kelly & A.C. Dietzsch, unpublished data).

The type locality, at 1333 m, is at the bottom of a deep, narrow valley, about 25–50 m from the bank of a small river. The site is riparian rain forest, dominated by tall trees, mainly *Liquidambar styraciflua* L. and *Cedrela odorata* L. The microclimate is moist and the vegetation lush and species-rich. The vicinity shows minor levels of disturbance: human disturbance due to the proximity of a seasonal camp-site with radiating trails, and natural disturbance due to wind-throw, and land-slips on the steeper slopes.

#### Etymology.

The name honors the Cusuco National Park in which it was found.

#### Discussion.

*Sommera cusucoana* differs from its Mesoamerican congeners by the combination of large, obovate leaves with long red petioles, glabrous or glabrate intervenal areas, red stipules, lax, sparsely pubescent 2–4-flowered inflorescences with red axes, flowers with red hypanthium and calyx, and long flowering and fruiting pedicels. Floral hypanthia and fruits of *Sommera cusucoana* are bright red at all developmental stages. Herbarium label notes for other *Sommera* species indicate fruits are green when immature and ripen red, at least in *Sommera chiapensis*, *Sommera donnell-smithii* and *Sommera montana*, and possibly white in *Sommera grandis* (Mexico). Only *Sommera chiapensis* Brandegee (Chiapas, Guatemala, Honduras) has flowers with similarly short, broadly triangular calyx lobes 1–2 mm long, but it differs in having shorter petioles 2–5 cm long, cymes with (4–)6–12 flowers, shorter corollas with tube 4–8 mm long, and smaller fruits 5–9 mm in diameter.

### Updated key to the Mesoamerican *Sommera* species

**Table:** 

1	Leaves glabrous beneath or strigillose only on the costa, even when young.	
2	Leaves 3.5–10.5 cm long, 0.8–2.5 cm wide	**6. *Sommera parva***
2’	Leaves 7–29 cm long, 1.8–12 cm wide.	
3	Calyx lobes 2.5–8 mm long, ovate-lanceolate to oblanceolate	***Sommera arborescens* p. p.**
3’	Calyx lobes 1–2 mm long, semicircular to narrowly triangular	***Sommera chiapensis***
1’	Leaves pilose-strigillose, strigillose, or sericeous beneath, at least when young.	
4	Branchlets glabrous; petioles 4.5–9 cm long; inflorescences 2–4-flowered; pedicels 10–28 mm long; hypanthium and calyx tube glabrous	***Sommera cusucoana***
4’	Branchlets densely strigillose to sericeous; petioles 0.1–5 cm long; inflorescences 3–25-flowered; pedicels (0)1–10 mm long; hypanthium and calyx tube densely strigillose-villous to sericeous.	
5	Mature flowers with calyx lobes 2.5–8 mm long, calyx tube 0.5–1 mm long; pedicels 1–10 mm long; Mexico, Guatemala	***Sommera arborescens* p.p.**
5’	Mature flowers with calyx lobes 1–6 mm long, calyx tube 1–4 mm long; pedicels 0–4 mm long; Guatemala to Panama.	
6	Mature flowers with calyx tube 3–4 mm long	***Sommera montana***
6’	Mature flowers with calyx tube 0.6–2 mm long.	
7	Mature flowers with calyx lobes 2.5–6 mm long, calyx tube 1–2 mm; Guatemala	***Sommera guatemalensis***
7’	Mature flowers with calyx lobes 1–5 mm long, calyx tube 0.6–1 mm; Honduras, Nicaragua, Costa Rica and Panama	***Sommera donnell-smithii***

This is the third species new to science discovered in Cusuco National Park by the Operation Wallacea Forest Botany team, the others being the tree *Hondurodendron urceolatum* Ulloa et al. (Aptandraceae: new genus and species) (Ulloa Ulloa et al. 2010, Kelly et al. 2011) and the herb *Calathea carolineae* H. Kenn. (Marantaceae) (Kennedy 2012). The type specimens of *Calathea carolineae* and *Sommera cusucoana* were collected at the same locality.

Sadly, this type locality is within 0.5 km distance of areas of extensive clear-fell, deep within the National Park, that were logged in the period 2010–13. Although the range of *Sommera cusucoana* has yet to be established, this relatively conspicuous and distinctive species has not been noted elsewhere within the Park, and its proposed conservation status must be Critically Endangered (IUCN 2000, 2013).

## Supplementary Material

XML Treatment for Sommera cusucoana
